# Impact of methylene blue and atorvastatin combination therapy on the apparition of cerebral malaria in a murine model

**DOI:** 10.1186/1475-2875-12-127

**Published:** 2013-04-15

**Authors:** Jérome Dormoi, Sébastien Briolant, Camille Desgrouas, Bruno Pradines

**Affiliations:** 1 Unité de Parasitologie, Institut de Recherche Biomédicale des Armées, Marseille, France; 2 Unité de Recherche sur les Maladies Infectieuses et Tropicales Emergentes, Aix Marseille Université, UM 63, CNRS 7278, IRD 198, Inserm 1095, Marseille, France; 3 Direction Interarmées du Service de Santé, Cayenne, Guyane; 4 Laboratoire de Parasitologie, Institut Pasteur de la Guyane, Cayenne, Guyane; 5 UMR MD3, Aix Marseille Université, Institut de Recherche Biomédicale des Armées, Marseille, France

**Keywords:** Malaria, *Plasmodium*, Anti-malarial drug, *in vivo*, Resistance, Methylene blue, Atorvastatin

## Abstract

**Background:**

Proveblue®, a methylene blue dye that complies with European Pharmacopoeia and contains limited organic impurities and heavy metals of recognized toxicity, showed *in vitro* synergy against *Plasmodium falciparum* when combined with atorvastatin, an inhibitor of 3-hydroxy-3-methylglutaryl-Coenzyme A reductase. The objective of this study was to evaluate the *in vivo* efficacy of Proveblue® when combined with atorvastatin in a murine model of experimental cerebral malaria.

**Methods:**

Forty female C57Bl6/N mice were divided into four groups (control, atorvastatin 40 mg/kg for seven days, Proveblue® 10 mg/kg for five days and atorvastatin combined with Proveblue®), infected with *Plasmodium berghei* ANKA parasites by intraperitoneal inoculation and observed for 45 days.

**Results:**

Treatment with atorvastatin alone did not demonstrate an effect significantly different from no treatment (p = 0.0573). All the mice treated by atorvastatin alone died. Treatment with Proveblue® or a combination of Proveblue® and atorvastatin was significantly increased survival of cerebral malaria (p = 0.0011 and 0.0002, respectively). Although there was only one death in the atorvastatin and Proveblue® combination treatment group (10%) *versus* two deaths (22%) with Proveblue® treatment, the effect on cerebral malaria was not significant (p = 0.283).

**Conclusions:**

The present work demonstrated, for the first time, the high efficacy of Proveblue® in preventing cerebral malaria. Atorvastatin alone or in combination appears to possess limited use for preventing cerebral malaria. Combination of atorvastatin with lower doses of Proveblue® (<10 mg/kg/day) should be evaluated to show potential synergistic effects in cerebral malaria prevention.

## Background

In 2002, the World Health Organization (WHO) recommended that artemisinin-based combination therapy (ACT) should be used for all cases of uncomplicated malaria. Four years later, the WHO further recommended that artesunate should be deployed as the first-line treatment for severe malaria in adults and replace quinine due to its efficacy and better tolerance [1]. In 2010, a large-scale trial confirmed the potency of artesunate for treating severe malaria in children [2]. In 2011, WHO recommended artesunate as the first-line treatment for severe malaria. Several recent studies have reported clinical failures or extended parasite clearance times in Cambodia [3-5].

There is an urgent need for the discovery of new anti-malarial drugs and combination therapy. A combinatorial approach protects each drug from the development of resistance and reduces the overall transmission rate of malaria [6].

Methylene blue (MB) is an old anti-malarial drug that is no longer in use. In 1891, Guttmann and Ehrlich were the first to report the anti-malarial properties of a synthetic thiazine dye, methylene blue, when they described the clinical cure of two patients after oral administration of MB [7]. Cardamatis wrote in *Progrès Médical* that he had found MB to be very effective in the early stages of severe malaria cachexia in cases that were resistant to quinine [8]. MB has shown *in vitro* activity against *Plasmodium falciparum* strains [9,10] or isolates [11,12] and *in vivo* activity against *Plasmodium vinckei* and *Plasmodium yoelii* parasites [13,14].

Currently, there is no MB available globally that complies with European Pharmacopoeia. To date, the pharmaceutical use of MB has been stymied by contamination with organic impurities and heavy metals with recognized toxicity. Provence Technologies and its subsidiary, Provepharm, have conducted four years of research that resulted in the first European Pharmacopoeia-grade MB: Proveblue®. This drug was obtained from an innovative synthetic and heavy-metal-free pathway using pharmaceutical-grade reagents (patent application N°FR06/06330, which has been extended to the international PCT reference PCT/FR/2007/001193). The total concentration of metals, Azure B (the most important impurity in MB) and other impurities in Proveblue® is <20 ppm, <2%, <0.5%, respectively. Proveblue® has *in vitro* anti-malarial activity (mean IC_50_ = 3.62 nM) against 23 *P. falciparum* strains that are resistant to other anti-malarial drugs [15]. No significant association was found between the Proveblue IC_50_ and polymorphisms in the genes that are involved in quinoline resistance, such as *pfcrt*, *pfmdr1*, *pfmdr2*, *pfmrp* and *pfnhe-1*; furthermore, there is no significant association between the Proveblue IC_50_ and the copy numbers of *pfmdr1* and *pfmdr2*[15]. While Proveblue® has *in vitro* antagonistic effects when combined with chloroquine and additive effects when combined with desethylamodiaquine against nine *P. falciparum* strains, Proveblue® exhibited noticeable synergistic effects when combined with mefloquine and quinine and high synergistic effects when combined with dihydroartemisinin, the active metabolite of artemisinin derivatives [16].

Statins, the inhibitors of 3-hydroxy-3-methylglutaryl-Coenzyme A reductase (HMG-CoA reductase) and a family of lipid-lowering drugs, have *in vitro* anti-malarial properties [17,18]. Atorvastatin (AVA), like the other statins, is not a highly active blood schizonticidal anti-malarial drug with IC_50_ values ranged from 2.5 to 12 μM [17,18]. Its application in malaria chemotherapy would be for adjuvant treatment. Moreover, AVA improved the *in vitro* activity of mefloquine [19], quinine [20], dihydroartemisinin [21] and Proveblue® [16] at the plasma concentrations expected in clinical observations in patients taking 80 mg of AVA daily (0.1 to 0.5 μM) [22]. However, AVA used alone failed to prevent death from cerebral malaria or to affect the parasitaemia of infected mice [23]. AVA combined with mefloquine led to a significant delay in mouse death and had an effect on the onset of cerebral malaria symptoms [24].

The objective of the present work was to evaluate the *in vivo* efficacy of Proveblue® when combined with AVA in a murine model of experimental cerebral malaria. While animal models do not exactly reproduce human malaria, they nevertheless exhibit some similarities to human cerebral malaria, and the *Plasmodium berghei* ANKA rodent parasite model is generally accepted as valid for studying experimental cerebral malaria pathogenesis [25,26].

## Methods

### Mice and experimental cerebral malaria

Forty female C57Bl6/N mice, six to seven weeks old and weighing 18–22 g (Charles Rivers, France), were infected on day 0 (D0) with *P. berghei* ANKA parasites by intraperitoneal (ip) inoculation. The inoculum contained 10^5^ parasitized erythrocytes extracted from infected donor C57Bl6/N mice and diluted in 200 μl normal saline. All animals were pathogen-free and were housed under standard conditions, with unlimited access to food and water. All efforts were made to minimize animal suffering. All experiments adhered to French guidelines for animal research and were approved by the ethical committee of the Institut de Recherche Biomédicale des Armées - Antenne de Marseille (Number 2007–09).

Except for the control group (8 mice), mice were treated daily when parasitaemia reached 0.1% by ip injection with 40 mg/kg AVA for seven days (7 mice), 10 mg/kg Proveblue® for five days (9 mice) or 40 mg AVA for seven days combined with 10 mg/kg Proveblue® during five days (10 mice). AVA calcium salt was dissolved in 1% dimethyl sulfoxyde (vol/vol) in 0.9% NaCl. The solution of 1% dimethyl sulfoxyde (vol/vol) in 0.9% NaCl was ip administered to control mice (after previously evaluation of absence of effect in infected and non-infected mice in comparison with 0.9% NaCl). Proveblue® was dissolved in 0.9% NaCl.

Parasitaemia was determined daily using Giemsa-stained thin blood smears collected from the tail vein. Parasitaemia was calculated by the number of infected red blood cells per 3,000 erythrocytes if >1% cells were infected and per 10,000 erythrocytes if <1% cells were infected. The animals were under daily supervision for clinical signs, neurological symptoms and weight. Experimental cerebral malaria was diagnosed by clinical signs based on a simplified SHIRPA protocol [27] with at least two symptoms in at least two of the three different groups: 1) alteration of autonomous function (piloerection, defecation, urination, respiration rate); 2) alteration of muscle tone and strength (grip strength, body tone, limb tone, abdominal tone); and, 3) ataxia, paralysis (mono-, hemi-, para-, or tetraplegia), deviation of the head, convulsions and coma.

## Statistical analysis

The data were analysed using R software® (version 2.10.1). Survival analyses were performed by the Kaplan-Meier log rank test. The comparison of medians between multiple groups was analysed by the Kruskal-Wallis test. The comparison of medians between two groups was analysed by the Mann–Whitney test. A difference was considered significant when P-values were less than 0.05.

## Results

**Figure 1:**
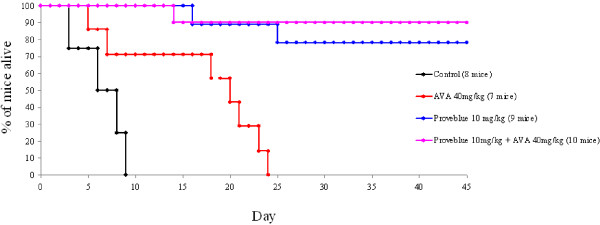
**Survival curve of C57Bl6/N mice infected on day 0 (D0) with *Plasmodium berghei *ANKA parasites and treated with 40 mg/kg atorvastatin (AVA) for seven days, 10 mg/kg Proveblue® for five days or 40 mg atorvastatin for seven days combined with 10 mg/kg Proveblue® for five days.**

In the control group, all the mice died before D10, with specific signs of cerebral malaria and parasitaemia <10.5% (6.7-10.5%) (Figure 1). The mice treated with 40 mg/kg AVA died between D5 and D9 (29% of the experimental group) with specific cerebral malaria symptoms and parasitaemia (5.7-6.8%) or between D18 and D24 with anaemia and parasitaemia (94.2-98.4%). In the group of mice treated with 10 mg/kg Proveblue®, one mouse (11%) had recurrent parasites at D5 and died at D16 (50.0% parasitaemia), and another had recurrent parasites at D11 and died at D25 (91.7% parasitaemia). These two mice died with no specific signs of cerebral malaria. After two days of treatment with Proveblue®, no parasite was detectable by blood smear. At D45, the surviving mice had no parasites. In the group of mice treated with 40 mg/kg AVA combined with 10 mg/kg Proveblue®, one mouse (10%) had recurrent parasites at D10 and died at D14 (6.6% parasitaemia) with specific signs of cerebral malaria. At D45, the surviving mice had no parasites.

## Discussion

Treatment with AVA alone did not demonstrate an effect significantly different from no treatment (p = 0.0573). All the mice treated by AVA alone died. These results confirm that in an experimental cerebral malaria model, therapeutic ip AVA treatment, similar to other statins, shows no effect on the incidence of cerebral malaria [23,24,28,29]. The pathogenesis of cerebral malaria in the murine model relies solely on the inflammatory response, unlike the pathogenesis of human cerebral malaria. Indeed, the cytoadherence phenomenon does not exist in *P. berghei* mice infections [30,31]. AVA strongly protects endothelial cell against *P. falciparum*-induced collateral damages, cell apoptosis and endothelial barrier permeabilization [32]. AVA can be used to reduce *P. falciparum* cytoadherence to endothelial cells; cytoadherence and the inflammatory burst are the key events of pathogenesis in severe human malaria. In mice with *P. berghei* ANKA cerebral malaria, lovastatin reduces pro-inflammatory cytokines in the brain and prevents cognitive impairment [33].

Treatment with Proveblue®, or a combination of Proveblue® and AVA, significantly reduced or prevented cerebral malaria (p = 0.0011 and 0.0002, respectively). A Proveblue® regimen of 10 mg/kg resulted in a 75% survival rate. This dosage is safe in children treated with methylene blue that was non-compliant with the European Pharmacopoeia [34,35]. Methylene blue at doses ranged from 12 mg/kg/day to 24 mg/kg/day for three days were administered to children with uncomplicated malaria in Burkina-Faso. Only one mouse died in the AVA and Proveblue® combination treatment group. The combination of Proveblue® and AVA was more effective than AVA alone in the experimental cerebral malaria mouse model (p = 0.0012). Although there was only one death with AVA plus Proveblue® treatment (10%) *versus* two deaths (22%) with Proveblue® alone, the effect on cerebral malaria was not significant (p = 0.283). Thus, the efficacy of the combination may be due to the effect of Proveblue®, not as a result of a synergism between the two compounds. The dose of 10 mg/kg of Proveblue® for five days, which showed a high efficacy when used alone, seems to be too high to show synergistic effects in combination with AVA. Combination with lower doses of Proveblue® should be evaluated. Proveblue® or AVA plus Proveblue® treatments are more effective than AVA and mefloquine combinations, which can delay mouse death and/or the onset of cerebral malaria but do not prevent death by anaemia [24]. The role of AVA in inhibiting cytoadherence in humans is not evaluable in mice, as this phenomenon does not exist in the *P. berghei* murine malaria model.

For the first time, the present work demonstrated the high efficacy of Proveblue® in preventing cerebral malaria. Another advantage of Proveblue® as an anti-malarial drug is that MB inhibits the maturation and transmission of gametocytes [36-38]. Of 44 compounds provided by Medicines for Malaria Venture, MB was the most active compound and reduced gametocyte viability with an IC_50_ value of 12 nM [39]. These results confirm the therapeutic potential of Proveblue®, a new MB formulation with limited organic impurities and toxic heavy metals that could be integrated into the pipeline of anti-malarial combination therapy [40]. AVA alone appears to possess limited use for preventing cerebral malaria. However, Proveblue® and AVA have different and complementary properties (effects on asexual stages, gametocyte maturation and transmission, cytoadherence, and pro-inflammatory cytokine levels), which makes them interesting partners for future investigation using combination therapy. Combination of AVA with lower doses of Proveblue® should be evaluated to show potential synergistic effects in cerebral malaria prevention. The present work demonstrated the high efficacy of Proveblue® in preventing cerebral malaria. The efficacy of Proveblue® and combination of AVA and Proveblue® should be evaluated in treating murine cerebral malaria.

## Competing interests

All authors declare that they have no potential conflicts of interest.

## Authors’ contributions

JD and CD carried out the *in vivo* study. SB and BP conceived and coordinated the study. JD and SB analysed the data. JD and BP drafted the manuscript. All the authors read and approved the final manuscript.

## Funding

This study was supported by the Délégation Générale pour l’Armement (grant no PDH-2-NRBC-4-B1-402).
